# Application of Synthetic Polymeric Scaffolds in Breast Cancer 3D Tissue Cultures and Animal Tumor Models

**DOI:** 10.1155/2017/8074890

**Published:** 2017-12-17

**Authors:** Girdhari Rijal, Chandra Bathula, Weimin Li

**Affiliations:** Department of Biomedical Sciences, Elson S. Floyd College of Medicine, Washington State University, Spokane, WA 99210, USA

## Abstract

Preparation of three-dimensional (3D) porous scaffolds from synthetic polymers is a challenge to most laboratories conducting biomedical research. Here, we present a handy and cost-effective method to fabricate polymeric hydrogel and porous scaffolds using poly(lactic-co-glycolic) acid (PLGA) or polycaprolactone (PCL). Breast cancer cells grown on 3D polymeric scaffolds exhibited distinct survival, morphology, and proliferation compared to those on 2D polymeric surfaces. Mammary epithelial cells cultured on PLGA- or PCL-coated slides expressed extracellular matrix (ECM) proteins and their receptors. Estrogen receptor- (ER-) positive T47D breast cancer cells are less sensitive to 4-hydroxytamoxifen (4-HT) treatment when cultured on the 3D porous scaffolds than in 2D cultures. Finally, cancer cell-laden polymeric scaffolds support consistent tumor formation in animals and biomarker expression as seen in human native tumors. Our data suggest that the porous synthetic polymer scaffolds satisfy the basic requirements for 3D tissue cultures both* in vitro* and* in vivo*. The scaffolding technology has appealing potentials to be applied in anticancer drug screening for a better control of the progression of human cancers.

## 1. Introduction

2D* in vitro* cell culture models have been instrumental in addressing various questions and providing invaluable knowledge in the field of cancer cell biology for decades. With the advancement of research technologies, some of the drawbacks of 2D cell culture models have been identified that include the lack of cell-ECM interactions and differences in cell morphology, proliferation rate, viability, polarity, motility, differentiation, and sensitivity to therapeutics compared to the characteristics of cells* in vivo* [1–6]. These limitations of 2D culture systems have become hindrance to the progress of our understanding of the mechanisms of cancer initiation and progression and of developing therapeutic approaches to treat human cancers, highlighting the needs for better culture platforms that are able to closely mimic tissue environments where native cancer cells live.

With the integration of the spatial concept, various 3D cell culture systems have been developed to overcome the limitations of 2D cultures. There is a remarkable increase in the use of 3D cultures over the past 10 years [7], resulting in many interesting findings that are distinct from the effects seen in the traditional 2D cultures. For instance, cells grown in 3D cultures display changes in metabolic characteristics, such as increased glycolysis [8], in gene expression patterns, such as upregulation of VEGF and angiopoietin genes involved in angiogenesis [9–11], and in production of chemokines, such as interleukin-8 [12], compared to cells grown on 2D surfaces. It is noteworthy that genome wide gene expression analysis comparing gene expression patterns of U87 cells grown in 2D and 3D cultures with a cohort of 53 pediatric high grade gliomas revealed significant similarities between the 3D, but not the 2D, culture samples and the human brain tumors [13]. Moreover, several studies have shown increased chemoresistance of cancer cells grown in 3D systems compared to the cells in 2D cultures [14–16], recapitulating the responses of cancer cells to chemotherapeutics* in vivo*. Depending on scientific questions to be addressed and specific experimental design, 3D scaffolds applied in biomedical research are predominantly fabricated using either natural materials, such as native tissue proteins and algae, or synthetic polymers, such as PLGA, PCL, and poly(ethylene glycol) (PEG) [7, 17, 18]. The advantages of synthetic polymeric scaffolds are their abundant availability, low cost, suitability for large-scale 3D bioprinting and reconstruction of certain tissue structures, and flexibility to be tailored to meet specific physical requirements of different culture systems [19–24].

In this study, we focused on characterizing the efficacies of applying the synthetic polymer scaffolds fabricated using PLGA and PCL with a modified gas foaming approach for 3D tissue cultures and animal models in breast cancer research. The viability, morphology, proliferation, and receptor expression of breast cancer cells as well as their responses to anticancer drug and development into tumors in animals with the support of the 3D scaffolds were investigated.

## 2. Materials and Methods

### 2.1. Polymer Coating on Slides

Microscopic glass slides were cleaned with 70% ethanol, air-dried in a biological safety cabinet, coated with 2% of PCL (Sigma-Aldrich), PLGA (Sigma-Aldrich), or PCL and PLGA (1 : 1 ratio) dissolved in chloroform (Sigma-Aldrich) for 1 hour, air-dried in a biological safety cabinet, and rinsed twice with 1x PBS before cell seeding.

### 2.2. 3D Porous Scaffold Fabrication from Polymer

To fabricate porous scaffolds with similar pore sizes as decellularized mouse breast tissues (~100 *μ*m) [16], 1.0 gram of PLGA or 0.5 gram of PCL was dissolved in 1 ml of chloroform followed by adding 1 gram of sodium bicarbonate (NaHCO_3_, Sigma-Aldrich) into the solution. The solutions were slowly dispensed into the semispherical molds (4 mm in diameter) built in porcelain panels, which were kept in −80°C freezer for 1-2 hours and freeze-dried at −50°C for 48 hours as described previously [16]. The scaffolds were then washed in 0.1 N hydrochloric acid (HCl) solution for 6 hours (replacing the solution hourly) at room temperature to generate the pores after releasing CO_2_ produced by the NaHCO_3_ and HCl reactions from the scaffolds, followed by washing in distilled water for several times until the pH of the water became neutral. The scaffolds were soaked in 70% ethanol for 3–5 hours, washed three times in 1x PBS for 10 minutes, and kept in 1x PBS until use. The generation of PCL and PLGA combined scaffolds was achieved by mixing equal volume (1 : 1 ratio) of PCL and PLGA solutions and following the same procedures as described above.

### 2.3. *In Vitro* 2D and 3D Cultures

MCF10A cells (American Type Culture Collection, ATCC) were maintained in 1x DMEM/F12 50/50 (Mediatech) supplemented with 10 *μ*g/ml insulin, 20 ng/ml EGF, 0.5 *μ*g/ml hydrocortisone, 100 ng/ml cholera toxin, 5% horse serum, and 1% Penicillin-Streptomycin. MDA-MB-231 cells (ATCC) were maintained in 1x DMEM (Mediatech) supplemented with 10% FBS and 1% Penicillin-Streptomycin. The polymer-coated slides (circular, 12 mm in diameter; ThermoFisher Scientific) or the scaffolds were placed in 24-well or 96-well plates, washed several times with sterile 1x PBS, and preconditioned with culture medium. MCF10A or MDA-MB-231 cells suspended in the respective culture medium were seeded on the slides or scaffolds (1 × 10^5^ per slide or scaffold) and allowed to attach to the matrices for 45 minutes. The cells were then cultured in the medium under optimal conditions (37°C, 5% CO_2_) and collected at indicated time points, analyzed, or used in downstream experiments. For longer period of culturing time, the culture medium was replenished every other day.

### 2.4. Live and Dead Cell Assay

The cell cultures on the polymer-coated slides or polymer scaffolds were briefly washed with 1x PBS (37°C) twice and incubated in the Live/Dead Cell Staining solution (2 *μ*M of calcein-AM and 4 *μ*M of EhtD-III in 1x PBS, PromoKine) at room temperature for 30 minutes. Images were captured using epifluorescence microscopy (Zeiss Axio Imager M2). Live cells take the calcein-AM stain and fluorescence green under EGFP filter, while dead cells take the EthD-III stain and fluorescence red under Texas Red filter.

### 2.5. Proliferation Assay

The proliferation of the cells grown on the coated slides and scaffolds was measured using the CCK-8 reagent (Sigma-Aldrich) at the time points indicated. Briefly, CCK-8 solution was added at a 1 : 10 dilution into the cultures and incubated (37°C, 5% CO_2_) for 3 hours. The supernatants of the cultures were collected and the colorimetric reactions within the supernatants that reflect the proliferation status were measured using a Synergy 2 microplate reader (BioTek) for the absorbance at 490 nm. Error bars represent standard deviations (SD) of the means of three independent experiments.

### 2.6. Cell Surface Receptor Expression

The cells cultured on the polymer-coated slides at about 80% confluency were washed with cold 1x PBS twice and fixed in 4% cold paraformaldehyde. Immunofluorescence staining was performed as previously described [25] using primary antibodies against integrin-*α*2 (mouse, Santa Cruz Biotechnology, sc-74466) and collagen type 1 (rabbit, Abcam, ab34710) as set 1 as well as integrin *α*6 (rabbit, Invitrogen, 710201) and laminin-*β*3 (mouse, Santa Cruz Biotechnology, sc-33178) as set 2 along with Alexa Fluor® dye-conjugated anti-rabbit and anti-mouse (Thermo Fisher Scientific) secondary antibodies to detect the expression of the integrin receptors on the surface of the cells in response to the polymer matrices.

### 2.7. Response of Cells to Anticancer Drug

T47D breast cancer cells (ATCC, 1 × 10^5^ cells per scaffold) were seeded on the 3D scaffolds and cultured in 96-well plates for 7 days. (Z)-4-Hydroxytamoxifen (4-HT, Abcam, ab1419430) at the final concentration of 1 *μ*M was administered in alternate days from 7th to 14th day of culture. Cell survival experiment was performed using the Live/Dead assay as described above. Triplicate independent experiments were performed for statistical significance.

### 2.8. *In Vivo* Tumor Formation

MDA-MB-231 cells (1 × 10^5^ cells/scaffold) were seeded on spherical porous PLGA scaffolds (4 mm-diameter) and cultured under optimal conditions (37°C, 5% CO_2_) for 24 hours prior to implantation. The blank (without cells as negative controls) and cell-laden scaffolds were implanted into the right and the left 4th inguinal mammary fat pads, respectively, of 8-week-old female NOD-SCID mice (Charles River Laboratories). Each implantation condition had six replicates. The growth of the tumors was monitored using spectrum computed tomography (CT) on an* in vivo* imaging system (IVIS, PerkinElmer). The tumors were collected into ice-cold 4% paraformaldehyde 4 weeks after implantation, paraffin embedded, cross-sectioned, antigen retrieved (1 mM EDTA solution, 10 mM Tris Base, and 0.05% Tween 20; pH 9.0), and stained with HER2 (rabbit, Cell Signaling Technology, 2165) and Ki-67 (mouse, Cell Signaling Technology, 9449) primary antibodies followed by Alexa fluorophore-conjugated secondary antibodies. Images were captured using fluorescence microscopy as described before [25].

### 2.9. Statistical Analysis

One-way ANOVA was performed using the StatPlus (Build 6.0.0/Core v5.9.92, AnalystSoft) software to analyze the statistical data. Error bars represent standard error of the mean (SEM) of three independent experiments unless otherwise indicated.

## 3. Results and Discussion

### 3.1. Cell Survival, Morphology, and Proliferation on the Polymeric Scaffolds

**Figure 1:**
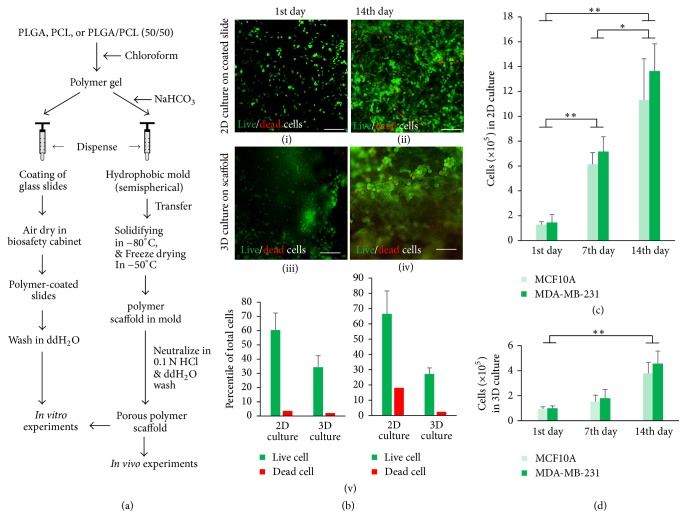
*Cell survival, morphology, and growth status on polymeric scaffolds*. (a) Main procedures of preparing PLGA-coated slides and 3D porous PLGA scaffolds. (b) Examination of MDA-MB-231 cell survival on PLGA-coated slides ((b)(i) and (b)(ii)) and porous PLGA scaffolds ((b)(iii) and (b)(iv)) using live and dead assays. Scale bars, 100 *μ*m. The number of live and dead cells in the 2D and 3D cultures were quantified in ((b)(v)). (c) Proliferation rate of MCF10A and MDA-MB-231 cells on PLGA-coated slides. (d) Proliferation rate of MCF10A and MDA-MB-231 cells on porous PLGA scaffolds. ^*∗*^*p* < 0.05, ^*∗∗*^*p* < 0.01.

To examine the survival of cancer cells grown on the polymeric substrata, human triple (ER, PR, and HER2 receptor) negative breast cancer MDA-MB-231 cells were cultured on PLGA-coated microscopic glass slides (2D) and porous PLGA scaffolds (3D), respectively, as described in the methods and illustrated in Figure 1(a) for 14 days. The Day 1 and Day 14 culture samples were collected and stained with the Live/Dead Cell assay kit as described in the methods. This staining method labels live cells in green color and the dead cells in red color when observing the cells under fluorescence microscope. Our results showed that the number of dead cells detected on PLGA-coated glass slides (Figures 1(b)(i) and 1(b)(v)) or on PLGA 3D scaffolds (Figures 1(b)(iii) and 1(b)(v)) were negligible on Day 1. However, the number of dead cells detected on the PLGA-coated glass slides was markedly higher (Figures 1(b)(ii) and 1(b)(v)) than those on the 3D PLGA porous scaffolds (Figures 1(b)(iv) and 1(b)(v)) on Day 14. The reason for increased cell death in the 2D cultures could be due to the faster proliferation rate of MDA-MB-231 cells on flat surface compared to that of the cells on 3D scaffolds, consistent with the previous observations where other scaffolding materials were used in 3D cell cultures [16, 26]. Because of the nature of the staining and imaging method, some of the cells grown on 3D scaffolds appeared to be out of focus due to the growth of cells at different focal planes/depths of the 3D scaffolds (Figure 1(b)(iii) and 1(b)(iv)).

We next inspected the morphological differences between cancer cells grown on polymeric 2D surfaces and those on polymeric 3D scaffolds. The results showed that the MDA-MB-231 cells grown on the 2D PLGA surfaces were in spindle shapes and populated the surface areas in a more or less universal way (Figure 1(b)(ii)) while those cultured on the 3D PLGA scaffolds exhibited round shapes and expand as cell clusters (Figure 1(b)(iv)). These data are consistent with previous studies that showed distinct morphological features of cells grown on 3D scaffolds compared to those of cells gown on 2D cultures [27–29]. The morphological differences between the two types of cultures could be the results of two factors. One is the distinct physical features of the surfaces of the 2D flat and 3D porous scaffolds, with the former being smooth and even and the latter being rough and uneven because of the existing pores on the surfaces of the scaffolds. The other is the spatial interactions between the neighboring cells and between the cells and the substrata, with the 2D interactions being “bidirectional” at lateral and basal surfaces of the cells and the 3D interactions being “multidirectional” at most or all of the surfaces of the cells. The multidirectional interaction feature of the 3D cell cultures resembles the characteristics of the closed environment of native tissues, where the living cells attach to and interact with the surrounding matrices or/and other cells at all directions. Moreover, even though cancer cells grown in 2D culture can grow on top of each other when the cell population reaches confluency, they hardly form tumoroids as can be commonly achieved in 3D cultures. Therefore, the morphological properties of cancer cells within 3D microenvironments could be a fundamental factor contributing to cancer cell growth, motility, tumor development, and resistance to therapeutic drugs.

Cancer cell proliferation after adapting the living environment is essential for tumor growth. To assess the support of PLGA in cell proliferation both in 2D and in 3D cultures, MCF10A and MDA-MB-231 cells were grown on the PLGA-coated glass slides and the porous PLGA scaffolds for 14 days. Cell proliferation rates were measured using CCK-8 reagent on Day 1, Day 7, and Day 14 of culture. The results showed that MCF10A and MDA-MB-231 cells grown on the PLGA-coated glass slides proliferated rapidly during the culturing time (Figure 1(c)). Though a similar trend was observed in the 3D cultures, the proliferation rate of the cells was substantially lower than that of the 2D cultures (Figures 1(c) and 1(d)). In addition, the proliferation rate of MDA-MB-231 cells is slightly higher than that of MCF10A cells in both 2D and 3D cultures (Figures 1(c) and 1(d)). A similar discrepancy in cell proliferation between 2D and 3D cultures was observed in our previous studies using native tissue ECM as scaffolding materials [16] and in studies using other materials [22, 30]. However, increased proliferation rate was observed in JIMT1 breast cancer cells grown on Matrigel compared to regular 2D cultures [31], suggesting a cell type- or/and culture system-related phenotype that should be taken into account for different experimental purposes. Overall, the proliferation rates of cell lines displayed in 3D culture models resemble those of tumor models* in vivo*.

### 3.2. Surface Receptor Expression of the Cells on Polymeric Scaffolds

**Figure 2:**
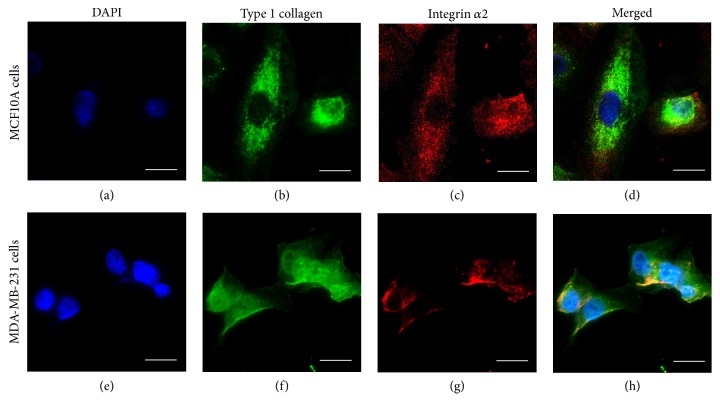
*Type I collagen and integrin 2 receptor expression in breast epithelial cells cultured on PLGA-coated surfaces*. The expression and deposition of type I collagen (green) as well as its cell surface receptor integrin *α*2 (red) expression was inspected in MCF10A and MDA-MB-231 cells grown on the PLGA-coated glass slides using IF staining couple with confocal microscopy. The nuclei of the cells were stained with DAPI (Blue). Scale bars, 10 *μ*m.

Type I collagen is one of the major components of breast tissue ECM [16] and integrin *α*2*β*1 is a primary receptor for type I collagen [32]. To investigate whether the synthetic polymers support the expression of ECM proteins and cell surface receptors, MCF-10A and MDA-MB-231 cells were cultured on the PLGA-coated slides for 24 hours, fixed with 4% paraformaldehyde, and stained with antibodies against type I collagen and integrin *α*2 as described previously [25]. Immunofluorescence (IF) microscopy detected strong staining signals of type I collagen and integrin *α*2 in both MCF10A and MDA-MB-231 cells (Figures 2(b), 2(c), 2(f), and 2(g)). Although the MDA-MB-231 cells appeared to be a bit smaller than the MCF10A cells on the PLGA-coated slides, the overall expression of type I collagen and integrin *α*2 in the cancer cells is lower than in the normal MCF10A cells (Figures 2(b)–2(d) and 2(f)–2(h)). These data are consistent with a previous report of lower integrin *α*2 (ITGA2) expression in primary breast cancers compared to normal breast tissues [33]. In addition, basal level expression of integrin *α*2*β*1 may favor breast cancer cell migration and tumor growth [34, 35] since high levels of the integrin receptor inhibit cancer cell migration [36]. We observed a nice colocalization of integrin *α*2 receptors with type I collagen in the cells especially around the edges of the cells, implicating local deposition of type I collagen on the slide surface and the binding of integrin *α*2 receptors to the deposited collagen for the attachment and migration of the cells. Similarly, we examined the expression levels of type I collagen and integrin *α*2 in MCF10A and MDA-MB-231 cells grown on PCL- or PLGA/PCL- (50/50) coated slides, and the results were coherent with those seen on the PLGA substratum (data not shown). Moreover, we did not notice significant differences in the morphology, viability, and cell proliferation of the cells grown on PLGA, PCL, or 3D PLGA/PCL (50/50) surfaces (data not shown). These data suggest that the synthetic polymeric surfaces or scaffolds could be used to study certain aspects of cancer biology. However, care needs to be taken into account in terms of the choices of different types of synthetic materials and fabrication methods to make 3D scaffolds for either biomedical or bioengineering applications owing to different advantages and limitations of the respective approaches compared to some biomaterial-based model systems [7].

### 3.3. Response of Cells Grown on the Polymeric Scaffold to Drugs

Traditionally, the efficacies of anticancer prodrugs were initially tested in 2D cell culture systems, and the promising drug candidates from these studies were further evaluated in animal experiments before entering clinical trials. However, the majority of drug candidates that were efficacious in 2D cultures failed in animal studies or clinical trials. One of the main reasons attributed to these drug testing failures is the inability of 2D culture systems to mimic the natural tissue microenvironment for cells living in them behave as they would be in native tissues. There is increasing evidence supporting 3D tissue cultures as better models to test efficacies of drug candidates [7].

**Figure 3:**
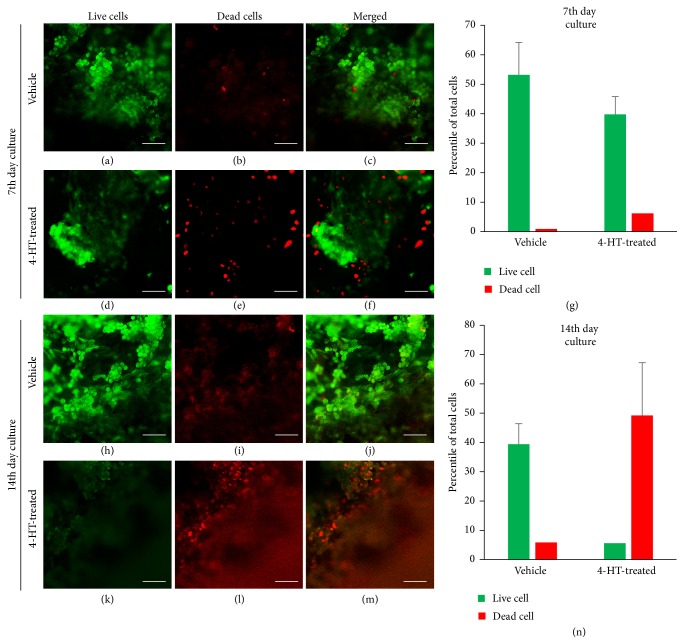
*Sensitivity of cancer cells grown on 3D PLGA scaffolds to anticancer drugs*. MDA-MB-231 cells cultured on the scaffolds for 7 days were treated with 4-HT every other day and examined for cell survival on Day 8 and Day 14, respectively. Live cells were indicated by green signals and dead cells in red signals. Scale bars, 100 *μ*m.

In this study, we examined the effect of 4-hydroxytamoxifen (4-HT), an active metabolite of the estrogen receptor (ER) antagonist tamoxifen, on the ER-positive luminal A type of breast cancer T47D cells grown on 3D PLGA scaffolds. The cells were treated with 1 *μ*M 4-HT on alternate days starting on Day 7 through Day 13 after cell seeding on the scaffolds, and the images were taken on Day 8 and Day 14 time points. The viability of the cells was analyzed using live and dead cell assays as described before. In consistency with the drug testing results seen in biomaterial-based 3D tissue culture studies [16] and animal models [37–39], T47D cells grown on 3D PLGA scaffolds were less sensitive to 4-HT than those on PLGA-coated slides (data not shown). T47D cells treated with vehicle solvent only showed increased cell proliferation on Day 14 compared to Day 7 and did not show significant differences in cell viability on Day 7 or Day 14 in cells grown on the 3D scaffolds (Figures 3(a)–3(c) and 3(h)–3(j)). However, the proliferation of T47D cells on the scaffolds was markedly decreased after 4-HT treatment as exhibited in the Day 7 and Day 14 images (Figures 3(d)–3(g) and 3(k)–3(n)). A close to complete cell death was observed in 4-HT-treated samples on Day 14 (Figures 3(k)–3(n)). These data collectively support the notion that cancer cells cultured in 3D environments develop chemoresistance as seen in native tumors and suggest that the polymeric scaffolds can be used as tissue-mimicry environments to study cancer cell responses to therapeutic drugs. The chemoresistance noticed in 3D cultures could be due to the heterogeneous populations of cancer cells and the complex physiochemical properties of the ECM environments deposited by the cells within the 3D structures that affect the permeability of the drugs and overexpression of multidrug resistance proteins [40–42].

### 3.4. The Polymeric Scaffold Support of Tumor Formation

**Figure 4:**
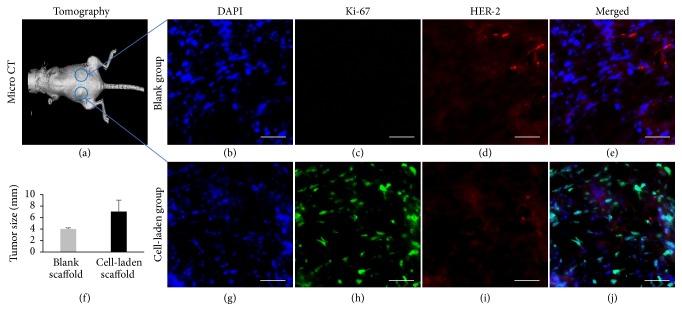
*Support of the polymeric scaffolds for tumor formation in mice*. Blank porous PLGA scaffolds (without MDA-MB-231 cells) and MDA-MB-231 cell-laden PLGA scaffolds were implanted into the mammary fat pads of the mice. Tumor growth were dynamically observed using IVIS during 4 weeks of the period ((a) and (f)). The cross sections of the tumors collected at the end point of the experiments were stained for DAPI (blue, (b) and (g)), Ki-67 (green, (c) and (h)), and HER2 (red, (d) and (i)). Scale bars, 100 *μ*m.

To assess the capabilities of the polymeric porous scaffolds in supporting tumor formation in mice, porous PLGA scaffolds were coated with MDA-MB-231 cells, cultured for 24 hours, and implanted into mammary fat pads of NOD/SCID mice. Blank scaffolds without cells were used as negative control. Tumor growth was monitored by an* in vivo* imaging system (IVIS) spectrum CT, and tumor sizes were measured with caliper. The tumors were collected 4 weeks after implantation, paraffin embedded, and cross-sectioned for immunohistochemistry (IHC) staining and analyzed with IF microscopy. The animal whole body tomographic images taken at the experimental end point demonstrated that tumor lumps were developed from the cancer cell-laden scaffolds, but not the blank scaffold control groups, during the period of observation (Figures 4(a) and 4(f)). The IF images showed that, by end of week 4 after implantation, the scaffolds were occupied by cells as illustrated by DAPI staining of nuclei of the cells on the cross sections of the samples (Figures 4(b) and 4(g)). As expected, the proliferating cell nuclear antigen biomarker Ki-67 was nondetectable in the blank scaffold implants and was detected at high levels within the tumors derived from the MDA-MB-231 cell-laden PLGA scaffolds (Figures 4(c) and 4(h)), indicating that fast proliferation of the cancer cell population was established in the scaffolds embedded within the native breast tissues. On the other hand, the HER2 receptors, which were negative in the MDA-MB-231 cells, were detected in some of the stromal cells within the normal and tumor tissues but not in the cancer cells (Figures 4(d) and 4(i)). We did not notice significant differences in tumor sizes when comparing the PLGA scaffold groups with the PCL or PLGA/PCL (50/50) scaffold groups in parallel animal experiments (data not shown). These data support the feasibility of applying the porous polymeric scaffolds in animal tumor model generation with the advantage of consistent tumor formation within reasonable period of time.

## 4. Conclusions and Remarks

3D cell cultures have overcome many limitations of 2D culture models in cancer biology studies. Our data have added further insights into how the synthetic polymer scaffolds can be successfully used in 3D tissue cultures and in animal tumor models. The phenotypes of the cancer cells we observed in the 3D cultures with regard to survival, morphology, proliferation, type I collagen and its receptor expression, and response to 4-HT treatment are very encouraging for additional research applications of the system in cancer research. For example, cancer cell migration and interaction with other types of cells within the 3D pores of the scaffolds can be studied. Because of the nonbiological features of the polymeric materials, nucleic acids and proteins can be extracted from the 3D cultures for further analysis without interference from biomolecules derived from native tissues.

Since the conventional tumor generation model, which injects cancer cells into the dorsal subcutaneous or mammary fat pads of animals, has big variations in tumor growth [43–45], our 3D porous scaffold-based animal tumor model can be very useful in consistently generating experimental tumors for both biomedical research and preclinical drug screening. Animal tumors produced using this scaffolding method can facilitate the observations of cancer biomarker expression, molecular regulation of cancer progression, and drug efficacies across tumors at similar sizes and developmental stages. Importantly, this easy and economically inexpensive scaffolding method could be adapted to bioengineering and other relevant fields. However, despite the rapid progress in the development of 3D culture models, there is not a one-for-all 3D system that could recapitulate all the features of native human tumors, and each model has its own advantages and disadvantages. Hence, it is important to select the 3D culture systems that best fit specific research purposes.
